# 3-Mesityl-2-oxo-1-oxaspiro­[4.4]non-3-en-4-yl benzoate

**DOI:** 10.1107/S1600536810021343

**Published:** 2010-06-16

**Authors:** Chuan-ming Yu, Zong-cheng Wang, Huan Zhou, Ming-hua Ji, Jin-hao Zhao

**Affiliations:** a College of Pharmaceutical Sciences, Zhejiang University of Technology, Hangzhou 310032, People’s Republic of China; b Research Center of Analysis and Measurement, Zhejiang University of Technology, Hangzhou 310032, People’s Republic of China; c Institute of Pesticide and Environmental Toxicology, Zhejiang University, Hangzhou 310029, People’s Republic of China

## Abstract

In the title compound, C_24_H_24_O_4_, a derivative of the potent insecticide and miticide spiro­mesifen, one cyclo­pentane C atom is disordered over two positions with occupancies of 0.574 (12) and 0.426 (12), resulting in respective envelope and twisted conformations for the cyclo­pentane ring. The atom at the flap position is 0.620 (5) Å out of the mean plane formed by the other four atoms of the envelope form. The furan ring makes dihedral angles of 68.26 (3) and 69.38 (2)°, respectively, with the 2,4,6-trimethyl­phenyl and benzene rings. The dihedral angle between the two benzene rings is 62.27 (3)°.

## Related literature

For the pesticide spiro­mesifen, the central unit of the title compound, see: Bayer Aktiengesellschaft (1995 ▶). For the synthesis and biological activity of spiro­mesifen derivatives, see: Ji *et al.* (2009 ▶); Zhao *et al.* (2009 ▶). For distance restraints, see: Watkin (1994 ▶).
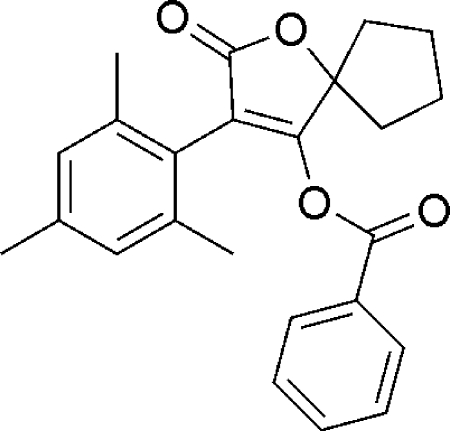


## Experimental

### 

#### Crystal data

C_24_H_24_O_4_
*M* *_r_* = 376.43
Monoclinic, 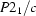

*a* = 8.4799 (5) Å
*b* = 15.9912 (9) Å
*c* = 15.9520 (8) Å
β = 106.240 (1)°
*V* = 2076.8 (2) Å^3^
*Z* = 4
Mo *K*α radiation
μ = 0.08 mm^−1^
*T* = 296 K
0.57 × 0.45 × 0.32 mm

#### Data collection

Rigaku R-AXIS RAPID diffractometer
Absorption correction: multi-scan (*ABSCOR*; Higashi, 1995 ▶) *T*_min_ = 0.955, *T*_max_ = 0.975
19157 measured reflections
4647 independent reflections
2622 reflections with *I* > 2σ(*I*)
*R*_int_ = 0.031

#### Refinement

*R*[*F* ^2^ > 2σ(*F* ^2^)] = 0.051
*wR*(*F* ^2^) = 0.164
*S* = 1.00
4647 reflections
267 parameters
22 restraints
H-atom parameters constrained
Δρ_max_ = 0.25 e Å^−3^
Δρ_min_ = −0.25 e Å^−3^

### 

Data collection: *PROCESS-AUTO* (Rigaku, 2006 ▶); cell refinement: *PROCESS-AUTO*; data reduction: *CrystalStructure* (Rigaku, 2007 ▶); program(s) used to solve structure: *SHELXS97* (Sheldrick, 2008 ▶); program(s) used to refine structure: *SHELXL97* (Sheldrick, 2008 ▶); molecular graphics: *ORTEP-3 for Windows* (Farrugia, 1997 ▶); software used to prepare material for publication: *WinGX* (Farrugia, 1999 ▶) and *PLATON* (Spek, 2009 ▶).

## Supplementary Material

Crystal structure: contains datablocks I, global. DOI: 10.1107/S1600536810021343/si2265sup1.cif

Structure factors: contains datablocks I. DOI: 10.1107/S1600536810021343/si2265Isup2.hkl

Additional supplementary materials:  crystallographic information; 3D view; checkCIF report

## Figures and Tables

**Fig. 1.**
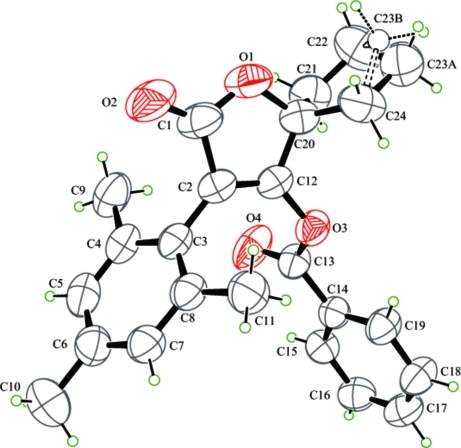
The molecular structure of (I), showing the atom-labelling scheme and the disordered CH2 group indicated as dashed lines. Displacement ellipsoids are drawn at the 50% probability level.

## supplementary crystallographic information

### Comment

4-hydroxyl-3-(2,4,6-trimethylphenyl)-1-oxaspiro[4,4]non-3-en-2-one (HTPO) is a key intermediate of Spiromesifen, which is an efficient insecticide and miticide, developed by Bayer Aktiengesellschaft (1995), see (Ji *et al.* 2009). As part of our continuing interest in the design and synthesis of the new insecticide and miticide, we have isolated the title compound (I), by the condensation reaction of benzoyl chloride and HTPO as colorless crystals. The molecule of the title compound (Fig. 1), exhibits a similar conformation and the same double bond characteristics as reported for the chlorobenzoate structure (Ji *et al.* 2009).

There was an indication of positional disorder in the crystal structure, detected with the checkCIF structure validation Program *PLATON* (Spek, 2009), which showed in the alert level B section significant Hirshfeld rigid bond test differences, 10.75 s.u. for C23—C24. Atom C23 was split into atoms C23A and C23B, which were refined using SIMU and PART instructions of *SHELXL97*, and four distance restraints were applied (Watkin, 1994). The C22—C23A, C22—C23B, C24—C23A and C24—C23B bond distance were restrained to 1.510 (2) Å, 1.514 (2) Å, 1.517 (2) Å and 1.5151 (19) Å, respectively.

The cyclopentane ring with C23B displays an envelope conformation with the C24 atom at the flap position 0.620 (5) Å out of the mean plane formed by the other four atoms, whereas the cyclopentane ring with C23A displays a twisted conformation, twisted on C21—C22.

### Experimental

4-hydroxyl-3-(2,4,6-trimethylphenyl)-1-oxaspiro[4,4]non-3-en-2-one (0.272 g, 1 mmol), 4-dimethylaminopyridine (0.012 g, 0.1 mmol), triethylamine (0.131 g, 1.3 mmol) and dry chloroform (10 ml) were added to a 25 ml round flask. Then the mixture was stirred and cooled to 273 K. Within 30 min benzoyl chloride (0.168 g, 1.2 mmol) was added dropwise to the solution at 273 K. After the reaction mixture was reacted at room temperature for 3 h, 1% HCl was added. The organic layer was washed to neutral with water and dried over Na_2_SO_4_ After filtered and concentrated, the organic residue was purified by silica gel column chromatography, eluted with ethyl acetate-petrum (1:3, *v*/*v*) to give a white solid (yield 83%, 0.312 g), which was then recrystallized from 95% ethanol to give colourless blocks.

### Refinement

H atoms were included in calculated positions and refined using a riding model, with C—H distances constrained to 0.96 Å for methyl H atoms, 0.93 Å for aryl H atoms and 0.98Å for the remainder, and with *U*_iso_(H) = 1.2*U*_eq_(C) or 1.5*U*eq(C) for methyl H atoms.

### Crystal data

**Table:**

C_24_H_24_O_4_	*F*(000) = 800
*M**_r_* = 376.43	*D*_x_ = 1.204 Mg m^−^^3^
Monoclinic, *P*2_1_/*c*	Mo *K*α radiation, λ = 0.71073 Å
Hall symbol: -P 2ybc	Cell parameters from 10851 reflections
*a* = 8.4799 (5) Å	θ = 3.1–27.4°
*b* = 15.9912 (9) Å	µ = 0.08 mm^−^^1^
*c* = 15.9520 (8) Å	*T* = 296 K
β = 106.240 (1)°	Chunk, colorless
*V* = 2076.8 (2) Å^3^	0.57 × 0.45 × 0.32 mm
*Z* = 4	

### Data collection

**Table:**

Rigaku R-AXIS RAPID diffractometer	4647 independent reflections
Radiation source: rolling anode	2622 reflections with *I* > 2σ(*I*)
graphite	*R*_int_ = 0.031
Detector resolution: 10.00 pixels mm^-1^	θ_max_ = 27.4°, θ_min_ = 3.1°
ω scans	*h* = −10→10
Absorption correction: multi-scan (*ABSCOR*; Higashi, 1995)	*k* = −20→17
*T*_min_ = 0.955, *T*_max_ = 0.975	*l* = −17→20
19157 measured reflections	

### Refinement

**Table:**

Refinement on *F*^2^	Secondary atom site location: difference Fourier map
Least-squares matrix: full	Hydrogen site location: inferred from neighbouring sites
*R*[*F*^2^ > 2σ(*F*^2^)] = 0.051	H-atom parameters constrained
*wR*(*F*^2^) = 0.164	*w* = 1/[σ^2^(*F*_o_^2^) + (0.0597*P*)^2^ + 0.8207*P*] where *P* = (*F*_o_^2^ + 2*F*_c_^2^)/3
*S* = 1.00	(Δ/σ)_max_ < 0.001
4647 reflections	Δρ_max_ = 0.25 e Å^−^^3^
267 parameters	Δρ_min_ = −0.25 e Å^−^^3^
22 restraints	Extinction correction: *SHELXL97* (Sheldrick, 2008), Fc^*^=kFc[1+0.001xFc^2^λ^3^/sin(2θ)]^-1/4^
Primary atom site location: structure-invariant direct methods	Extinction coefficient: 0.035 (3)

### Special details

**Table:**

Geometry. All e.s.d.'s (except the e.s.d. in the dihedral angle between two l.s. planes) are estimated using the full covariance matrix. The cell e.s.d.'s are taken into account individually in the estimation of e.s.d.'s in distances, angles and torsion angles; correlations between e.s.d.'s in cell parameters are only used when they are defined by crystal symmetry. An approximate (isotropic) treatment of cell e.s.d.'s is used for estimating e.s.d.'s involving l.s. planes.
Refinement. Refinement of *F*^2^ against ALL reflections. The weighted *R*-factor *wR* and goodness of fit *S* are based on *F*^2^, conventional *R*-factors *R* are based on *F*, with *F* set to zero for negative *F*^2^. The threshold expression of *F*^2^ > σ(*F*^2^) is used only for calculating *R*-factors(gt) *etc*. and is not relevant to the choice of reflections for refinement. *R*-factors based on *F*^2^ are statistically about twice as large as those based on *F*, and *R*- factors based on ALL data will be even larger.

### Fractional atomic coordinates and isotropic or equivalent isotropic displacement parameters (Å^2^)

**Table:**

	*x*	*y*	*z*	*U*_iso_*/*U*_eq_	Occ. (<1)
O3	0.59902 (17)	0.68318 (9)	0.43446 (9)	0.0620 (4)	
O4	0.8496 (2)	0.73853 (11)	0.45217 (12)	0.0889 (6)	
O1	0.3476 (2)	0.84301 (10)	0.30647 (12)	0.0840 (5)	
O2	0.3938 (3)	0.83890 (14)	0.17466 (12)	0.1114 (8)	
C14	0.8250 (2)	0.61060 (12)	0.52516 (12)	0.0512 (5)	
C3	0.6519 (3)	0.70257 (13)	0.24733 (13)	0.0598 (5)	
C19	0.7173 (3)	0.54964 (13)	0.53702 (14)	0.0639 (6)	
H19	0.6052	0.5551	0.5106	0.077*	
C15	0.9908 (3)	0.60287 (13)	0.56565 (13)	0.0579 (5)	
H15	1.0632	0.6440	0.5583	0.070*	
C13	0.7675 (3)	0.68381 (13)	0.46840 (13)	0.0566 (5)	
C12	0.5273 (3)	0.73987 (13)	0.37080 (13)	0.0577 (5)	
C8	0.6291 (3)	0.61735 (14)	0.22738 (13)	0.0605 (5)	
C7	0.7338 (3)	0.57817 (15)	0.18622 (14)	0.0668 (6)	
H7	0.7190	0.5216	0.1728	0.080*	
C2	0.5435 (3)	0.74710 (13)	0.29100 (14)	0.0624 (6)	
C17	0.9423 (3)	0.47286 (15)	0.62716 (15)	0.0714 (6)	
H17	0.9821	0.4257	0.6606	0.086*	
C5	0.8798 (3)	0.70432 (17)	0.18587 (15)	0.0743 (7)	
H5	0.9642	0.7334	0.1720	0.089*	
C18	0.7768 (3)	0.48087 (15)	0.58814 (16)	0.0734 (7)	
H18	0.7048	0.4398	0.5962	0.088*	
C16	1.0493 (3)	0.53393 (15)	0.61723 (14)	0.0668 (6)	
H16	1.1609	0.5289	0.6451	0.080*	
C20	0.4067 (3)	0.79956 (14)	0.38972 (15)	0.0645 (6)	
C4	0.7791 (3)	0.74694 (15)	0.22727 (14)	0.0678 (6)	
C6	0.8588 (3)	0.62051 (16)	0.16464 (15)	0.0710 (6)	
C1	0.4252 (3)	0.81278 (16)	0.24846 (17)	0.0796 (7)	
C21	0.4808 (3)	0.86271 (16)	0.46176 (17)	0.0774 (7)	
H21A	0.5716	0.8379	0.5058	0.093*	
H21B	0.5206	0.9115	0.4378	0.093*	
C9	0.8105 (4)	0.83721 (16)	0.25219 (18)	0.0909 (8)	
H9A	0.9100	0.8550	0.2402	0.136*	
H9B	0.8210	0.8438	0.3133	0.136*	
H9C	0.7204	0.8705	0.2190	0.136*	
C11	0.4958 (3)	0.56714 (16)	0.24929 (17)	0.0778 (7)	
H11A	0.5270	0.5551	0.3107	0.117*	
H11B	0.4801	0.5157	0.2170	0.117*	
H11C	0.3954	0.5986	0.2342	0.117*	
C24	0.2621 (3)	0.76212 (15)	0.4166 (2)	0.0859 (8)	
H24A	0.1634	0.7648	0.3679	0.103*	
H24B	0.2842	0.7039	0.4326	0.103*	
C10	0.9716 (4)	0.5763 (2)	0.1205 (2)	0.1052 (10)	
H10A	0.9949	0.5211	0.1443	0.158*	
H10B	1.0722	0.6072	0.1303	0.158*	
H10C	0.9193	0.5726	0.0589	0.158*	
C22	0.3449 (4)	0.8866 (2)	0.5007 (2)	0.1037 (10)	
H22A	0.2826	0.9332	0.4690	0.124*	
H22B	0.3895	0.9027	0.5613	0.124*	
C23A	0.2365 (12)	0.8105 (4)	0.4935 (5)	0.095 (3)	0.426 (12)
H23A	0.1223	0.8266	0.4829	0.115*	0.426 (12)
H23B	0.2690	0.7774	0.5465	0.115*	0.426 (12)
C23B	0.1941 (4)	0.8384 (3)	0.4509 (5)	0.091 (2)	0.574 (12)
H23C	0.1270	0.8716	0.4033	0.109*	0.574 (12)
H23D	0.1283	0.8222	0.4892	0.109*	0.574 (12)

### Atomic displacement parameters (Å^2^)

**Table:**

	*U*^11^	*U*^22^	*U*^33^	*U*^12^	*U*^13^	*U*^23^
O3	0.0500 (9)	0.0678 (9)	0.0656 (9)	0.0025 (7)	0.0116 (7)	0.0185 (7)
O4	0.0676 (11)	0.0851 (12)	0.1018 (13)	−0.0220 (9)	0.0034 (9)	0.0370 (10)
O1	0.0854 (13)	0.0734 (11)	0.0842 (11)	0.0284 (9)	0.0089 (9)	0.0066 (9)
O2	0.1274 (18)	0.1159 (16)	0.0777 (13)	0.0473 (14)	0.0069 (11)	0.0328 (11)
C14	0.0498 (12)	0.0531 (11)	0.0494 (10)	−0.0009 (9)	0.0120 (8)	0.0015 (8)
C3	0.0631 (14)	0.0602 (13)	0.0522 (11)	0.0049 (10)	0.0097 (9)	0.0130 (9)
C19	0.0525 (13)	0.0618 (13)	0.0733 (14)	−0.0042 (10)	0.0110 (10)	0.0110 (10)
C15	0.0499 (12)	0.0638 (13)	0.0577 (12)	−0.0023 (10)	0.0111 (9)	−0.0029 (9)
C13	0.0493 (12)	0.0626 (13)	0.0546 (11)	−0.0041 (10)	0.0090 (9)	0.0063 (9)
C12	0.0559 (13)	0.0528 (12)	0.0594 (12)	0.0028 (9)	0.0081 (9)	0.0076 (9)
C8	0.0583 (13)	0.0627 (13)	0.0573 (12)	0.0021 (10)	0.0110 (10)	0.0091 (9)
C7	0.0689 (15)	0.0656 (14)	0.0642 (13)	0.0049 (11)	0.0159 (11)	0.0052 (10)
C2	0.0634 (14)	0.0563 (12)	0.0613 (13)	0.0079 (10)	0.0074 (10)	0.0083 (9)
C17	0.0786 (17)	0.0606 (14)	0.0675 (14)	0.0115 (12)	0.0083 (12)	0.0103 (10)
C5	0.0707 (16)	0.0876 (18)	0.0652 (14)	−0.0082 (13)	0.0197 (12)	0.0191 (12)
C18	0.0730 (16)	0.0621 (14)	0.0813 (16)	−0.0060 (12)	0.0152 (12)	0.0175 (11)
C16	0.0598 (14)	0.0698 (14)	0.0634 (13)	0.0083 (11)	0.0050 (10)	0.0021 (10)
C20	0.0617 (14)	0.0572 (12)	0.0703 (14)	0.0025 (10)	0.0114 (11)	−0.0010 (10)
C4	0.0781 (16)	0.0649 (14)	0.0572 (13)	−0.0026 (12)	0.0135 (11)	0.0158 (10)
C6	0.0692 (16)	0.0805 (17)	0.0647 (14)	0.0046 (13)	0.0209 (11)	0.0103 (11)
C1	0.0821 (18)	0.0734 (16)	0.0718 (16)	0.0207 (13)	0.0028 (13)	0.0111 (12)
C21	0.0757 (17)	0.0659 (15)	0.0873 (17)	−0.0098 (12)	0.0176 (13)	−0.0127 (12)
C9	0.118 (2)	0.0694 (16)	0.0866 (18)	−0.0153 (15)	0.0303 (16)	0.0134 (13)
C11	0.0752 (17)	0.0737 (16)	0.0869 (17)	−0.0075 (13)	0.0268 (13)	−0.0015 (12)
C24	0.0620 (16)	0.0648 (15)	0.132 (2)	−0.0049 (12)	0.0293 (15)	−0.0144 (15)
C10	0.095 (2)	0.121 (3)	0.115 (2)	0.0060 (19)	0.0548 (19)	−0.0022 (19)
C22	0.091 (2)	0.122 (3)	0.095 (2)	0.0028 (19)	0.0220 (16)	−0.0362 (18)
C23A	0.096 (3)	0.096 (3)	0.095 (3)	−0.0014 (10)	0.0283 (13)	0.0004 (10)
C23B	0.089 (2)	0.090 (2)	0.093 (2)	0.0003 (10)	0.0265 (11)	−0.0017 (10)

### Geometric parameters (Å, °)

**Table:**

O3—C12	1.370 (2)	C16—H16	0.9300
O3—C13	1.379 (2)	C20—C24	1.530 (3)
O4—C13	1.191 (2)	C20—C21	1.527 (3)
O1—C1	1.365 (3)	C4—C9	1.501 (3)
O1—C20	1.458 (3)	C6—C10	1.512 (4)
O2—C1	1.207 (3)	C21—C22	1.503 (4)
C14—C15	1.379 (3)	C21—H21A	0.9700
C14—C19	1.385 (3)	C21—H21B	0.9700
C14—C13	1.477 (3)	C9—H9A	0.9600
C3—C4	1.401 (3)	C9—H9B	0.9600
C3—C8	1.400 (3)	C9—H9C	0.9600
C3—C2	1.482 (3)	C11—H11A	0.9600
C19—C18	1.378 (3)	C11—H11B	0.9600
C19—H19	0.9300	C11—H11C	0.9600
C15—C16	1.382 (3)	C24—C23B	1.5151 (19)
C15—H15	0.9300	C24—C23A	1.517 (2)
C12—C2	1.323 (3)	C24—H24A	0.9700
C12—C20	1.490 (3)	C24—H24B	0.9700
C8—C7	1.392 (3)	C10—H10A	0.9600
C8—C11	1.505 (3)	C10—H10B	0.9600
C7—C6	1.380 (3)	C10—H10C	0.9600
C7—H7	0.9300	C22—C23A	1.510 (2)
C2—C1	1.480 (3)	C22—C23B	1.514 (2)
C17—C18	1.372 (3)	C22—H22A	0.9700
C17—C16	1.372 (3)	C22—H22B	0.9700
C17—H17	0.9300	C23A—H23A	0.9700
C5—C6	1.382 (4)	C23A—H23B	0.9700
C5—C4	1.395 (3)	C23B—H23C	0.9700
C5—H5	0.9300	C23B—H23D	0.9700
C18—H18	0.9300		
			
C12—O3—C13	118.83 (16)	O1—C1—C2	109.5 (2)
C1—O1—C20	109.94 (17)	C22—C21—C20	106.1 (2)
C15—C14—C19	119.88 (19)	C22—C21—H21A	110.5
C15—C14—C13	118.49 (18)	C20—C21—H21A	110.5
C19—C14—C13	121.62 (18)	C22—C21—H21B	110.5
C4—C3—C8	120.4 (2)	C20—C21—H21B	110.5
C4—C3—C2	118.9 (2)	H21A—C21—H21B	108.7
C8—C3—C2	120.7 (2)	C4—C9—H9A	109.5
C18—C19—C14	119.7 (2)	C4—C9—H9B	109.5
C18—C19—H19	120.1	H9A—C9—H9B	109.5
C14—C19—H19	120.1	C4—C9—H9C	109.5
C14—C15—C16	120.0 (2)	H9A—C9—H9C	109.5
C14—C15—H15	120.0	H9B—C9—H9C	109.5
C16—C15—H15	120.0	C8—C11—H11A	109.5
O4—C13—O3	121.64 (19)	C8—C11—H11B	109.5
O4—C13—C14	127.1 (2)	H11A—C11—H11B	109.5
O3—C13—C14	111.30 (17)	C8—C11—H11C	109.5
C2—C12—O3	128.8 (2)	H11A—C11—H11C	109.5
C2—C12—C20	113.61 (18)	H11B—C11—H11C	109.5
O3—C12—C20	117.45 (18)	C23B—C24—C20	101.7 (3)
C7—C8—C3	118.7 (2)	C23A—C24—C20	109.5 (2)
C7—C8—C11	119.3 (2)	C23B—C24—H24A	85.6
C3—C8—C11	122.1 (2)	C23A—C24—H24A	109.8
C6—C7—C8	122.2 (2)	C20—C24—H24A	109.8
C6—C7—H7	118.9	C23B—C24—H24B	138.0
C8—C7—H7	118.9	C23A—C24—H24B	109.8
C12—C2—C1	105.3 (2)	C20—C24—H24B	109.8
C12—C2—C3	130.94 (19)	H24A—C24—H24B	108.2
C1—C2—C3	123.8 (2)	C6—C10—H10A	109.5
C18—C17—C16	120.4 (2)	C6—C10—H10B	109.5
C18—C17—H17	119.8	H10A—C10—H10B	109.5
C16—C17—H17	119.8	C6—C10—H10C	109.5
C6—C5—C4	122.4 (2)	H10A—C10—H10C	109.5
C6—C5—H5	118.8	H10B—C10—H10C	109.5
C4—C5—H5	118.8	C21—C22—C23A	106.5 (3)
C17—C18—C19	120.1 (2)	C21—C22—C23B	106.7 (3)
C17—C18—H18	119.9	C21—C22—H22A	110.4
C19—C18—H18	119.9	C23A—C22—H22A	110.4
C17—C16—C15	119.8 (2)	C23B—C22—H22A	82.0
C17—C16—H16	120.1	C21—C22—H22B	110.4
C15—C16—H16	120.1	C23A—C22—H22B	110.4
O1—C20—C12	101.61 (17)	C23B—C22—H22B	134.0
O1—C20—C24	110.1 (2)	H22A—C22—H22B	108.6
C12—C20—C24	117.10 (19)	C22—C23A—C24	104.5 (2)
O1—C20—C21	109.55 (19)	C22—C23A—H23A	110.8
C12—C20—C21	114.51 (19)	C24—C23A—H23A	110.8
C24—C20—C21	103.96 (19)	C22—C23A—H23B	110.8
C5—C4—C3	118.2 (2)	C24—C23A—H23B	110.9
C5—C4—C9	120.6 (2)	H23A—C23A—H23B	108.9
C3—C4—C9	121.1 (2)	C22—C23B—C24	104.4 (2)
C7—C6—C5	118.0 (2)	C22—C23B—H23C	110.9
C7—C6—C10	121.1 (3)	C24—C23B—H23C	110.9
C5—C6—C10	120.8 (2)	C22—C23B—H23D	110.9
O2—C1—O1	121.5 (2)	C24—C23B—H23D	110.9
O2—C1—C2	129.0 (3)	H23C—C23B—H23D	108.9
			
C15—C14—C19—C18	−1.1 (3)	C2—C12—C20—C21	−116.3 (2)
C13—C14—C19—C18	177.7 (2)	O3—C12—C20—C21	67.0 (3)
C19—C14—C15—C16	0.7 (3)	C6—C5—C4—C3	−0.3 (3)
C13—C14—C15—C16	−178.18 (19)	C6—C5—C4—C9	177.6 (2)
C12—O3—C13—O4	9.7 (3)	C8—C3—C4—C5	1.0 (3)
C12—O3—C13—C14	−170.80 (17)	C2—C3—C4—C5	−179.77 (19)
C15—C14—C13—O4	0.7 (3)	C8—C3—C4—C9	−176.9 (2)
C19—C14—C13—O4	−178.1 (2)	C2—C3—C4—C9	2.3 (3)
C15—C14—C13—O3	−178.75 (18)	C8—C7—C6—C5	0.6 (3)
C19—C14—C13—O3	2.4 (3)	C8—C7—C6—C10	179.6 (2)
C13—O3—C12—C2	63.9 (3)	C4—C5—C6—C7	−0.5 (4)
C13—O3—C12—C20	−119.9 (2)	C4—C5—C6—C10	−179.4 (2)
C4—C3—C8—C7	−0.9 (3)	C20—O1—C1—O2	179.0 (3)
C2—C3—C8—C7	179.92 (19)	C20—O1—C1—C2	−0.7 (3)
C4—C3—C8—C11	178.9 (2)	C12—C2—C1—O2	−177.9 (3)
C2—C3—C8—C11	−0.3 (3)	C3—C2—C1—O2	2.1 (5)
C3—C8—C7—C6	0.1 (3)	C12—C2—C1—O1	1.7 (3)
C11—C8—C7—C6	−179.8 (2)	C3—C2—C1—O1	−178.3 (2)
O3—C12—C2—C1	174.2 (2)	O1—C20—C21—C22	93.4 (3)
C20—C12—C2—C1	−2.1 (3)	C12—C20—C21—C22	−153.2 (2)
O3—C12—C2—C3	−5.8 (4)	C24—C20—C21—C22	−24.2 (3)
C20—C12—C2—C3	177.9 (2)	O1—C20—C24—C23B	−77.9 (3)
C4—C3—C2—C12	−111.4 (3)	C12—C20—C24—C23B	166.7 (3)
C8—C3—C2—C12	67.8 (3)	C21—C20—C24—C23B	39.3 (3)
C4—C3—C2—C1	68.6 (3)	O1—C20—C24—C23A	−109.0 (5)
C8—C3—C2—C1	−112.2 (3)	C12—C20—C24—C23A	135.6 (5)
C16—C17—C18—C19	1.4 (4)	C21—C20—C24—C23A	8.2 (5)
C14—C19—C18—C17	0.0 (4)	C20—C21—C22—C23A	31.8 (5)
C18—C17—C16—C15	−1.8 (4)	C20—C21—C22—C23B	−0.5 (4)
C14—C15—C16—C17	0.8 (3)	C21—C22—C23A—C24	−25.9 (8)
C1—O1—C20—C12	−0.5 (2)	C23B—C22—C23A—C24	69.0 (3)
C1—O1—C20—C24	−125.3 (2)	C23B—C24—C23A—C22	−69.3 (3)
C1—O1—C20—C21	121.0 (2)	C20—C24—C23A—C22	10.7 (8)
C2—C12—C20—O1	1.7 (2)	C21—C22—C23B—C24	25.4 (6)
O3—C12—C20—O1	−175.02 (17)	C23A—C22—C23B—C24	−69.1 (3)
C2—C12—C20—C24	121.7 (2)	C23A—C24—C23B—C22	68.8 (3)
O3—C12—C20—C24	−55.1 (3)	C20—C24—C23B—C22	−39.8 (5)
